# Inferring the Relative Resilience of Alternative States

**DOI:** 10.1371/journal.pone.0077338

**Published:** 2013-10-11

**Authors:** David G. Angeler, Craig R. Allen, Carmen Rojo, Miguel Alvarez-Cobelas, María A. Rodrigo, Salvador Sánchez-Carrillo

**Affiliations:** 1 Department of Aquatic Sciences and Assessment, Swedish University of Agricultural Sciences, Uppsala, Sweden; 2 Nebraska Cooperative Fish and Wildlife Research Unit, United States Geological Survey, School of Natural Resources, University of Nebraska-Lincoln, Lincoln, Nebraska, United States of America; 3 Cavanilles Institute for Biodiversity and Evolutionary Biology, University of Valencia, Valencia, Spain; 4 National Museum of Natural History, Spanish National Research Council, Madrid, Spain; Dauphin Island Sea Lab, United States of America

## Abstract

Ecological systems may occur in alternative states that differ in ecological structures, functions and processes. Resilience is the measure of disturbance an ecological system can absorb before changing states. However, how the intrinsic structures and processes of systems that characterize their states affects their resilience remains unclear. We analyzed time series of phytoplankton communities at three sites in a floodplain in central Spain to assess the dominant frequencies or “temporal scales” in community dynamics and compared the patterns between a wet and a dry alternative state. The identified frequencies and cross-scale structures are expected to arise from positive feedbacks that are thought to reinforce processes in alternative states of ecological systems and regulate emergent phenomena such as resilience. Our analyses show a higher species richness and diversity but lower evenness in the dry state. Time series modeling revealed a decrease in the importance of short-term variability in the communities, suggesting that community dynamics slowed down in the dry relative to the wet state. The number of temporal scales at which community dynamics manifested, and the explanatory power of time series models, was lower in the dry state. The higher diversity, reduced number of temporal scales and the lower explanatory power of time series models suggest that species dynamics tended to be more stochastic in the dry state. From a resilience perspective our results highlight a paradox: increasing species richness may not necessarily enhance resilience. The loss of cross-scale structure (i.e. the lower number of temporal scales) in community dynamics across sites suggests that resilience erodes during drought. Phytoplankton communities in the dry state are therefore likely less resilient than in the wet state. Our case study demonstrates the potential of time series modeling to assess attributes that mediate resilience. The approach is useful for assessing resilience of alternative states across ecological and other complex systems.

## Introduction

Ecological resilience is most simply defined as the amount of disturbance a system can tolerate without changing its original structure, processes, functions and feedbacks [1]. When critical thresholds are exceeded, ecological systems can undergo a regime shift; that is, they are pushed to an alternative state with new structures, functions and processes [2-4]. Despite ecological patterns and processes differing between alternative states, the resilience that emerges from these distinct patterns and processes may not necessarily differ between states. In practice this means that alternative states can be as resilient as prior states and resist returning to a state that existed prior to a regime shift (hysteresis) [5]. Understanding ecological patterns and processes that affect the resilience of alternative states is critical [6]. However, the complex nature of ecological systems, including multidimensional, hierarchical and nonlinear phenomena, has hindered the objective assessment of resilience of alternative states to disturbances in ecological systems. In this paper, we use time series modeling to identify attributes of resilience (within and cross scale structure) of alternative states. We demonstrate the potential of the approach using phytoplankton community dynamics in alternative states of a semiarid floodplain.

Theory and empirical research suggest that the dynamic structure of ecosystems is controlled by a small set of ecological processes that operate at characteristic temporal and spatial scales [7-10]. Biological interactions entrain community assembly relatively quickly at local spatial (habitat) scales. Biogeographical processes influence communities over regional spatial and paleoecological temporal scales. Phylogenetic factors are mainly evident over spatially large scale domains with slow dynamics. A multi-scale spatiotemporal perspective of ecosystems is useful because resilience emerges from a reinforcement of structures and processes (i.e. feedbacks) that operate within and across scales [11,12]. For instance, community change in subarctic lakes has been shown to be scale-specific, with a subgroup of littoral invertebrates tracking slow changes of regional environmental conditions, while other subgroups responded to faster-changing processes that were unrelated to environmental change [13]. In this example, resilience emerges from cross-scale reinforcement of structures.

Tools have been developed that allow quantification and comparison of sudden changes in ecological patterns (i.e. discontinuity analyses [14]). That is, the ability to measure and quantify discontinuities provides insight regarding the number of dominant scales of process and structure that are present in a system [15], and can therefore serve as a means to assess cross-scale structures that can be important for reinforcing processes across scales in specific states of ecological systems. Multivariate time series modeling can identify temporal structure in data sets [16,17]. It identifies different temporal frequency patterns in the abundance or biomass structure of communities. The temporal patterns and frequency structure that can be discerned have upper bounds set by the limit of the temporal extent of the data series, and lower bounds set by the frequency of sample collection. It allows an assessment of the dynamic compositional structure in ecological communities that most likely arises from, and thus reflect, processes that steer community dynamics in a specific state. An advantage of time series modeling is that it captures patterns of dynamic structure without the need to understand the full range of causality [18]. Because the method allows tracking temporal variability within a time scale (e.g. decadal, inter-annual or seasonal) and among these time scales, we are able to infer the temporal cross-scale structure by assessing community dynamics at different temporal scales [17]. 

Multivariate time series modeling can test for the presence of temporal frequencies in species abundance and biomass, and the prevailing periods of these cycles. We can then determine the distribution of species within and across these temporal scales, to provide a tool to assess resilience. For instance, species richness explains the resilience of ecosystems [19,20] but this may not be generalizable across systems because species evenness patterns rather than richness can also influence resilience [21]. Time series modeling can untangle the importance of richness mediating resilience because it explicitly accounts for a compartmentalization of diversity by scale; it also allows for an assessment of the distribution of functional groups within and across these scales, which is relevant for understanding resilience [11,13]. For example, only a few dominant species might explain fluctuation frequencies at a few temporal scales detected in a hypothetical system state A, relative to a system state B where both species richness within scales and the number of scales is higher. The resilience of state B is likely higher, not only because of a higher cross-scale structure, but also a higher within-scale redundancy of patterns (species distributions within temporal scales), suggesting a stronger reinforcement of functions and processes, strengthening the feedbacks that maintain systems in a specific state. 

In this study, we examine both within and cross scale distributions of species to assess aspects of relative resilience of alternative states. We study these resilience aspects in a floodplain wetland in semiarid Spain as a model system. Wetlands are ecosystems that are strongly regulated by hydrological disturbance, which in turn is regulated by climate [22]. In dryland countries, the variability between wet and dry alternating phases can be very pronounced [23], causing changes in habitat availability, water quality, thermal stress, trophic conditions, resource competition, and the importance of stochastic versus deterministic community assembly processes [24-27]. Although alternating wet and dry phases characterize the seasonal disturbance regime at the ecosystem and landscape scale [28], climate change increases the magnitude, frequency and duration of extreme wet and dry spells [29,30], especially in arid countries [31]. These transitions can be non-linear [32], triggering state shifts in hydrological functioning and biological communities [33-36]. Thus, non-linear abiotic and biotic changes triggered by climatic extreme events suggest that ecosystems can shift between alternative wet and dry states on supraseasonal time scales. These state shifts need to be discerned from the seasonally recurring wet-dry phases. The increased likelihood of state shifts in ecosystems resulting from global change is increasingly recognized [19,37].

In this paper we study resilience characteristics in this context. We discerned an abrupt transition of a supraseasonal wet period resulting from extreme precipitation events (1997, 1998) to a period of prolonged drought (2000, 2001) from seasonally recurring wet-dry phases in the wetland. The non-linear change of hydroperiods identified highlights the presence of two distinct abiotic states in the wetland for which substantial differences in abiotic and biotic conditions have been shown (overview in [38]). Hydroperiod and water dynamics critically control plankton structure, function and processes in wetlands and shifts in hydrological condition inevitably lead to a profound re-organization of phytoplankton communities [39,40]. We assessed community dynamics and resilience attributes of phytoplankton (resilience of what [41]) to the ecological conditions within these wet and dry states (resilience to what). 

 Ideally, alternative equilibria can be established as such after a complete community turnover [42] (but see 43). Phytoplankton fulfills this criterion in the context of our study, which is comprised of two-years of wet and dry periods. Phytoplankton have short generation times (days to weeks [44]) and their dynamics capture relevant ecological processes during these periods. Therefore, phytoplankton are ideal organisms to assess alternative equilibria (alternative states) over the two-years of wet and dry periods of our study. The main study hypothesis of this paper is that patterns and processes of phytoplankton community dynamics differ between wet and dry states in the floodplain, reflecting the reorganization of communities due to changes in the driving processes of hydroperiods and flooding regimes. These differences should be manifested in the within-scale (species richness compartmentalized by scale) and cross-scale (temporal scales of fluctuations patterns) structure of phytoplankton community dynamics between both states. Using phytoplankton dynamics in the floodplain for demonstration, we expect that the time series modeling approach used here can be used in other studies to assess the relative resilience of alternative states in ecological and other complex systems.

## Materials and Methods

### Ethics statement

All field sampling and laboratory analyses reported in this study complied with national and international standards, and was authorized by the Spanish national park authority. The field studies did not involve endangered or protected species.

### Site description

**Figure 1:**
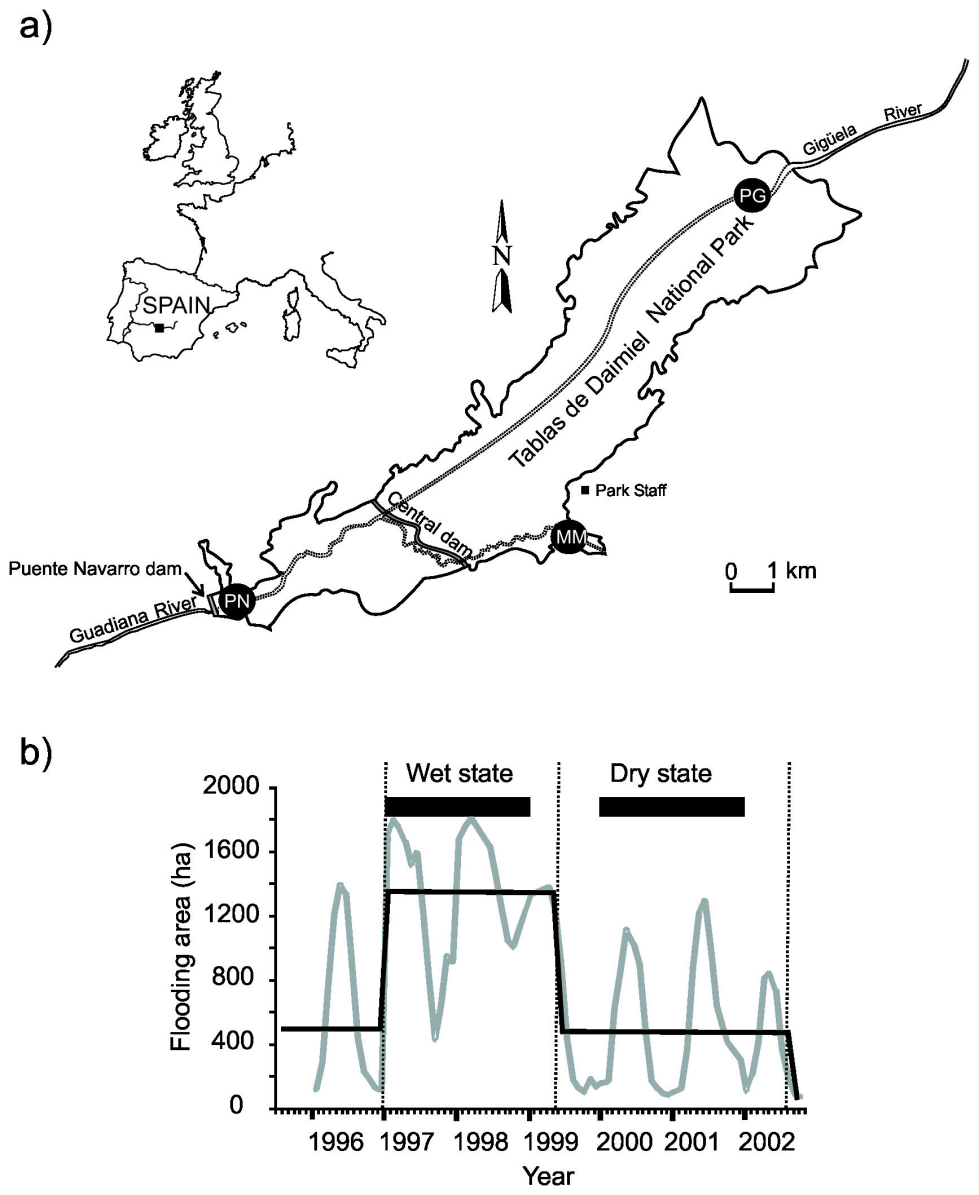
Location of the Las Tablas de Daimiel floodplain wetland, a National Park in central Spain (a), and clearly discernable hydrological states, revealed by regime shift detection (b). Shown are the patterns of flooded area (grey line) between 1996 and 2003, state transitions (black line), and the periods covered for analyses of phytoplankton community structure (horizontal black bars) in the wet and dry state.

Las Tablas de Daimiel National Park is a 1675-ha floodplain wetland (0.91 m average depth) situated in the Guadiana River watershed in central Spain (39°08’N, 3°43’W; Figure 1). We studied phytoplankton community dynamics at three sites (designated PG, MM, PN) that are highly idiosyncratic in terms of abiotic and biotic characteristics. PG is dominated by emergent macrophytes and fluctuates between a shallow pond or channelized system with riverine flow depending on the timing of major flooding events in the wetland. MM is a shallow turbid pond with some vegetation cover and a highly variable hydroperiod. PN is a deeper site with more stable hydroperiods resulting from damming (Figure 1a). Community dynamics were studied during two-year periods in contrasting wet and dry states (Figure 1b). These periods have been identified by means of the STARS (Sequential t-test analysis of regime shifts) algorithm, a method used for detecting regime shifts in univariate time series [45], using flooded area as a surrogate for climate-induced changes in ecological patterns and processes (see Appendix S1). Further information about this wetland can be found in [38].

### Sampling

Biovolume of phytoplankton taxa was measured at monthly intervals at PG, MM and PN during contrasting hydrological periods (wet state, 1997 and 1998; dry state, 2000 and 2001; Figure 1b), resulting in equidistant time series of 24 months per site and state. Given this sampling resolution we were able to model temporal frequency patterns from monthly to inter-annual (2 years) scales in both states. The PG site could not be sampled on several occasions during the wet state because flooding limited access. No time series modeling could therefore be made for this site for the wet state. Lugol-fixed phytoplankton samples were measured, counted, and taxonomic composition evaluated following [46]. Their biovolume (mm^3^ L^-1^) was calculated following the methods described in [47].

### Statistical analyses

*Community metrics* – We first assessed metrics that are commonly used in community ecology to characterize the phytoplankton community during the wet and dry states. Total biovolume, taxon richness (“richness”), and the Shannon-Wiener diversity index based on biovolume distributions among taxa were calculated for each sampling month per site and state (wet *vs* dry) using the software Primer 6 (Primer-E Ltd, Plymouth, UK). The Shannon-Wiener index was transformed into its number equivalents by exponentiation to make richness and diversity trends comparable [48]; the exponentiated Shannon-Wiener index will be considered as a measure of “diversity” that reflects different ecological processes from “richness” [49]. Evenness was calculated by dividing diversity with richness (evenness = diversity/richness); this achieves mathematical independence of evenness from richness [50].

 Repeated measures analysis of variance (rm-ANOVA) was conducted using Statistica v.5 (Statsoft Inc, Tulsa, OK, USA) to test for differences in these community metrics between the wet and dry state. We tested for the effects of “State” (wet *vs* dry), “Time” (24 sampling months in each state) and their interactions, using the sites (PG, MM, and PN) as independent replicates. “State” and “Time” comprised the independent variables while the community metrics comprised the dependent variables in the analysis. All data were log-transformed when necessary prior to analyses to fulfill the requirements of parametric tests. Because sphericity assumptions were violated in some comparisons, degrees of freedom (d.f.) were adjusted [51] to obtain more accurate significance levels (note that d.f. can be expressed as decimals as a result of this correction procedure). Inference was made at P < 0.05. Because our design was unbalanced due to the lack of PG samples during the wet state, we calculated rm-ANOVA models based on Type III sums of squares. We consider significant interaction terms between “State” x “Time” crucial for inferring differences in phytoplankton community metrics between the wet and dry state. If the studied metrics of phytoplankton community structure differ between states, these terms will be significant. 

*Time series modeling* – To assess patterns and scales of phytoplankton fluctuations, we constructed time series models based on redundancy analysis (RDA) [16]. We used temporal variables extracted by AEM analysis (Asymmetric Eigenvector Maps, [52-54]). Details of all steps in the analyses are in Appendix S2. Briefly, the AEM analysis produces a set of orthogonal temporal variables that are derived from the linear time vector that comprises the length of the study period (i.e., 24 equidistant time steps that comprise the temporal window for phytoplankton community dynamics in each state; wet and dry) and that can be used as explanatory variables to model temporal relationships in community data. The type of AEM variables computed in the present study was designed for spatial analysis to account for linear trends in the response variables. Because time comprises a directional process, AEM is more suitable for modeling linear trends relative to other methods (Principal Coordinates of Neighbor Matrices, PCNM; Moran Eigenvector Maps, MEM; [52-54]). This procedure yielded a total of 12 AEM variables with positive eigenvalues, each of which corresponds to a specific temporal structure and scale in the phytoplankton dynamics: the first AEM variable models linear trends and the subsequent variables capture temporal variability from slow to increasingly shorter fluctuation frequencies in the community data over the 24-month study period in each hydrological state (see 52; Appendix S2). Note that in our study the shortest fluctuation frequency captures monthly variation given that phytoplankton has been sampled once per month. For each state (wet, dry), we constructed a parsimonious temporal model for phytoplankton community dynamics at each site by running a forward selection on the AEM variables. Because AEM analysis is efficient in covering linear trends no detrending of models was necessary. 

The RDA retains significant AEM variables and these are linearly combined in ways to extract temporal patterns from the Hellinger-transformed species matrices; that is, the RDA identifies species with similar temporal patterns in the species time matrix and uses their temporal patterns to calculate a modeled species group trend for these species based on linearly combined AEMs. The significance of the temporal patterns of all modeled fluctuation patterns of species groups revealed by the RDA is tested by means of permutation tests. The RDA relates each modeled temporal fluctuation pattern with a significant canonical axis. The R software generates linear combination (lc) score plots, which visually present the modeled temporal patterns of species groups that are associated with each canonical axis. Counting the number of significant canonical axes, the cross-scale aspect of community dynamics important for resilience can be quantified. 

All relevant steps in the analyses are carried out with two functions implemented in R 2.15.1 statistical software package [55]. First, the conversion of the linear time vector to AEM variables is done using the “aem.time” function (AEM package). This function accounts for the connectivity of linear time steps, and a connectivity matrix, which needs to be calculated in spatial analysis, especially in hierarchical or dendritic designs [52-54], is therefore not necessary in time series analysis. All remaining steps (calculation of modeled species group trends, visual presentation of the results in form of lc score plots) are carried out with the “quickPCNM” function (PCNM package). The calculations are therefore based exclusively on an automatic statistical procedure, thereby avoiding potential researcher-induced bias in model construction. 

The scale-specific relevance of taxon richness, i.e. the within-scale aspect of resilience, was evaluated using correlation analyses. We used Spearman rank correlation analyses, relating the raw biovolume data of individual phytoplankton taxa with the modeled species group patterns, to assess scale-specific taxon richness. We also evaluated the number of species with presumably stochastic dynamics (that is, those that were not associated with any significant canonical axis) by subtracting the sum of species that correlated with canonical axes from the total number of species used for time series modeling for each site (PG, MM, PN) and state (wet, dry). In and of itself, discerning between species that explain the dominant temporal frequencies from stochastic species in a system is critical, because it allows us to separate patterns of cyclic change from stochastic noise, providing a more refined view of the contribution of species richness to within and cross scale reinforcement of processes (feedbacks) and thus resilience. 

## Results

### Hydrological patterns

Clear seasonal patterns in flooded area, with high flooded area in spring and lower water levels in summer, autumn and winter, were discerned between 1996 and 2002, showing the typical seasonally recurrent wet-dry phases in semiarid wetlands (Figure 1b). However, two distinct hydrological periods were identified on a supraseasonal scale, based on threshold detection using the STARS algorithm, one associated with very high water levels in the floodplain from 1997 to 1999 (wet state) and a second with low water levels during a supraseasonal drought (dry state) starting approximately by mid 1999 (Figure 1b). 

### Temporal patterns of community metrics

**Figure 2:**
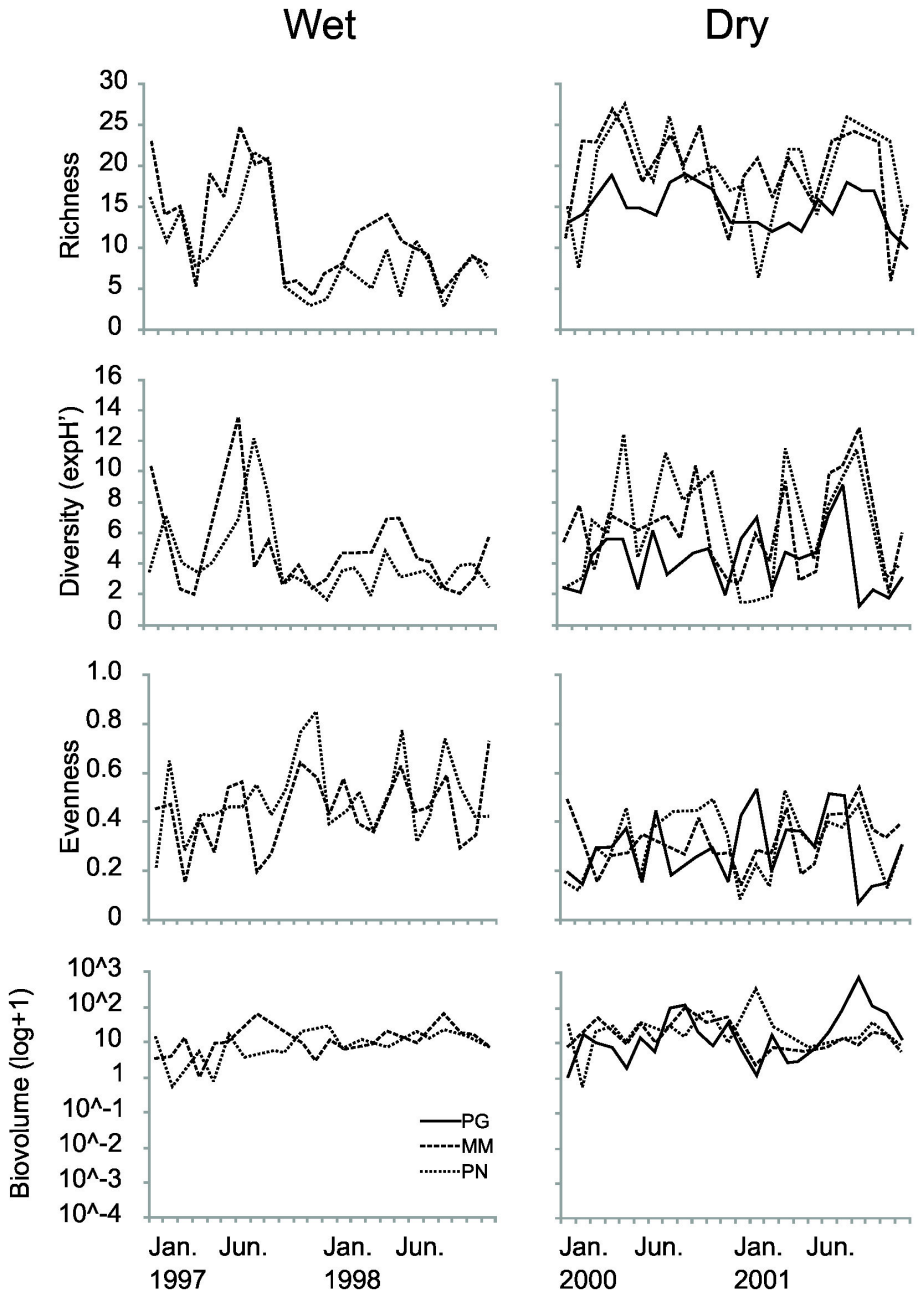
Temporal patterns of phytoplankton community metrics (taxon richness, exponentiated Shannon-Wiener diversity, evenness, and total biovolume) for the three study sites in the wet (left column) and dry (right column) state.

Based on community metrics (richness, diversity, evenness and total biovolume), different temporal patterns were present in the wet and dry states (Figure 2). Richness and diversity had different temporal patterns during both states, with higher between-year variability in the wet state (higher in 1997; lower in 1998) compared to the dry state where fluctuation patterns were similar between both years of study (Figure 2). On average, richness and diversity were higher whilst evenness was lower during the dry compared to the wet state (Figure 2), highlighting a different phytoplankton community structure between states. A significant interaction term was found for richness, diversity and evenness metrics (Table 1), highlighting that sites differ in their average richness, diversity and evenness and in their temporal patterns during both states (Figure 2). Total biovolume had similar temporal patterns across sites and these were not significantly different between both states (Table 1).

**Table 1:** Results of repeated measures ANOVA contrasting univariate community metrics (richness, diversity, evenness, total biovolume) between states (wet *vs* dry), time (24 months) and their interactions.

Metrics	Statistics	States	Time	States x Time
Richness	d.f.	**1, 3**	**7.49, 22.46**	**7.49, 22.46**
	MS	**1499.34**	**53.68**	**48.27**
	F	**11.58**	**3.89**	**3.50**
	P	**0.042**	**0.006**	**0.01**
Diversity	d.f.	1, 3	**23, 69**	**23, 92**
	MS	31.59	**9.953**	**11.772**
	F	1.144	**1.798**	**2.126**
	P	0.363	**0.033**	**0.007**
Evenness	d.f.	**1, 3**	**18.81, 56.43**	**15.86, 63.44**
	MS	**0.789**	**0.03**	**0.03**
	F	**51.65**	**2.003**	**2.072**
	P	**0.006**	**0.023**	**0.018**
Total biovolume	d.f.	1, 3	2.92, 8.77	2.92, 8,77
	MS	0.438	0.834	0.224
	F	1.099	1.121	0.302
	P	0.372	0.391	0.999

Shown are degrees of freedom (d.f.; Huynh-Feldt corrected), mean squares (MS), sources of variation (F), and significance levels (P). Significant terms are highlighted in bold.

### Time series modeling

**Figure 3:**
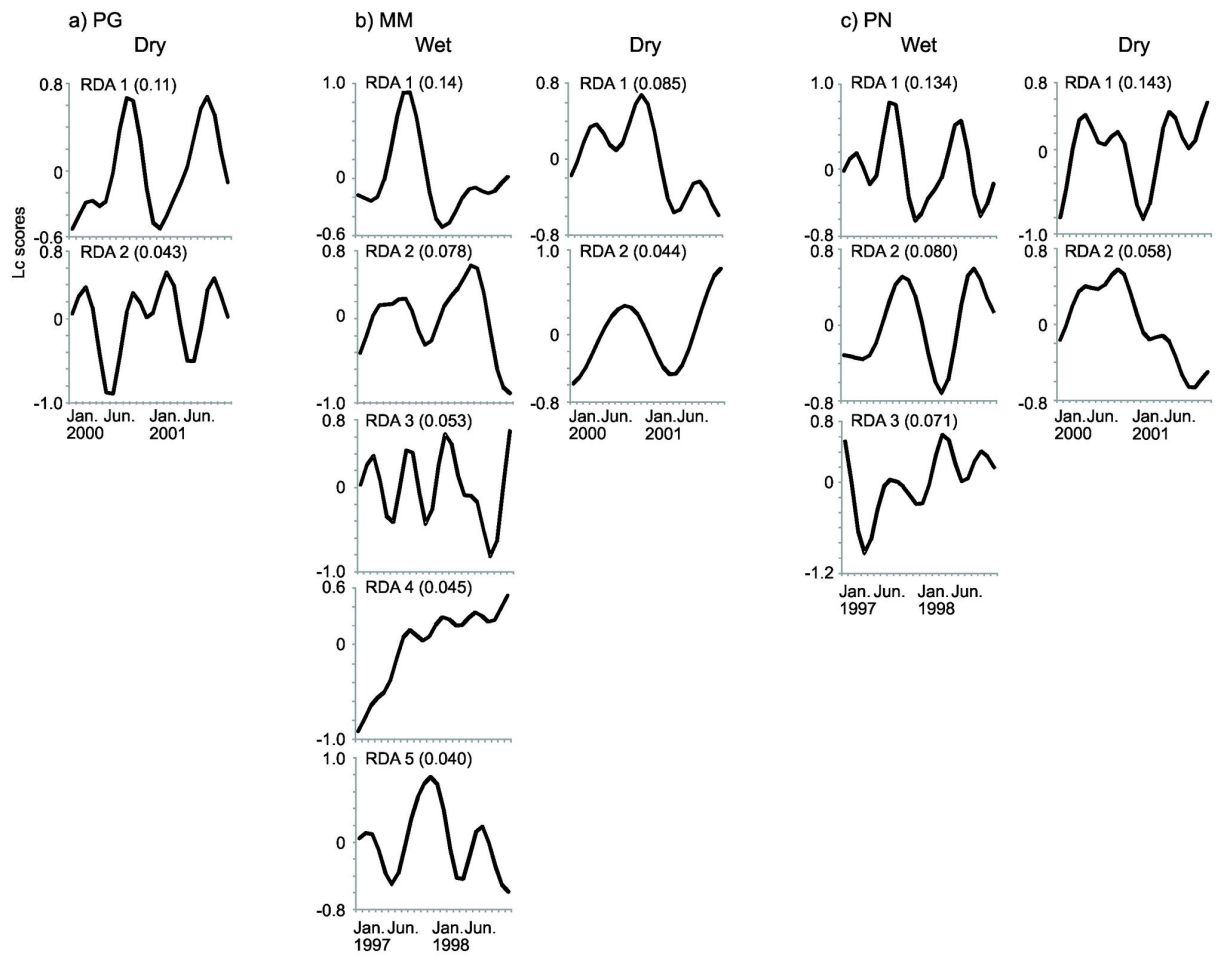
Time series models showing modeled fluctuation frequencies of groups of phytoplankton species at different temporal scales (RDA axes) at the three study sites (a, PG; b, MM, c, PN) during the wet and dry states. Shown are the patterns based on linear combination (Lc) scores of significant Asymmetric Eigenvector Maps (AEM) variables. The explanatory power of each axis (adjusted R^2^) is given in parentheses.

Assessing the temporal structure of phytoplankton in both states using time series modeling, we found contrasting patterns of scale-specific variability across the sites. During the wet state, the temporal dynamics of phytoplankton at the MM and PN sites showed diverse patterns of temporal variability, manifested in 5 (MM) and 3 (PN) distinct frequency patterns during the 24-month period. This identified groups of species within the phytoplankton community that had fluctuation patterns at distinct temporal scales (Figure 3). These scales usually covered between-year variability at canonical axes 1 and 2 (RDA 1 and RDA 2, Figure 3) that indicate slower temporal dynamics at these scales, and elements of faster change reflected by a stronger component of within-year variability at the remaining axes during the 24-months period (Figure 3). No clear patterns in the number of species contributing to these patterns (i.e. within-scale distribution of species) between states could be discerned (Table 2). The fluctuation patterns associated with RDA 1 were explained by 15 (MM) and 3 (PN) species in the wet state; in the dry state it was 14 (PG), 8 (MM) and 3 (PN) species. RDA 2 patterns were explained by 6 (MM) and 10 (PN) species in the wet state and 4 (PG), 5 (MM) and 10 (PN) species in the dry state. No comparisons could be made for the other canonical axes because these were not significant in the models of the dry state (see below; Table 2).

**Table 2:** Number of species correlating with significant canonical (RDA) axes revealed by Spearman rank correlation analyses, and the number of species that were not significantly correlated with any RDA axis (“Stochastic”).

	Wet state MM	PN	Dry state PG	MM	PN
RDA 1	15(30)	3(8)	14(19)	8(11)	3(4)
RDA 2	6(12)	10(26)	4(6)	5(7)	10(14)
RDA 3	2(4)	2(5)	---	---	---
RDA 4	8(16)	---	---	---	---
RDA 5	4(8)	---	---	---	---
Stochastic	15(30)	24(62)	44(61)	59(82)	68(94)
Total	50	39	62	72	81

Shown are also the total number of species in the communities (“Total”). Values in parentheses show percentages from that total. Values are given only for RDA axes that were significant (see Figure 3).

 Time series modeling also revealed different patterns of temporal structure between both states (Figure 3). All sites had temporal fluctuation patterns at two temporal scales in the dry state. For the MM and PN sites this means a decrease in temporal diversity patterns in the dry relative to the wet state (i.e., a lower cross-scale structure). Slow temporal patterns, reflecting mainly between-year variability characterized the dominant phytoplankton dynamics across site, while shorter-term fluctuations (within-year variability) were less important in the dry relative to the wet period. This is also reflected in the individual AEM variables retained in the RDA models after forward selection; that is, variables indicating faster patterns of change (e.g., AEM 9) were not selected for the dry state in MM and PN (Appendix S3). 

 Differences between the wet and dry states were also evident in terms of the strength of the reduced RDA models that provide insight into the relative importance of deterministic *vs* stochastic processes mediating community assembly. The explanatory power of the RDA models, derived from all significant and insignificant RDA axes, was higher for MM (adjusted R^2^ = 0.391) and PN (0.369) in the wet state, reflecting a higher deterministic component in community dynamics. In the dry state the strength of models decreased in MM (0.196) and PN (0.264), and in PG it was 0.217, suggesting increasing stochasticity. This trend was also reflected in the percentage of species that did not correlate significantly with the temporal frequency patterns identified in MM and PN (Table 2).

## Discussion

Alternative states theory posits that the ecological structure, functions and processes differ between states (e.g., [4]). Early warning statistics have been developed in recent years to assess when the resilience of one state erodes and a shift to another state occurs [56-59]. However, an estimation of the resilience of alternative states that emerges from the structure, functions and processes that define these states has remained elusive and methodologically challenging. We used time series modeling to reveal patterns of fluctuation frequencies of phytoplankton at independent temporal scales in contrasting wet-dry states of a semiarid floodplain. In turn, the patterns of within-scale (species richness compartmentalized by scale) and cross-scale (temporal scales of fluctuations patterns) structure identified allowed for an assessment of critical components of resilience and provided insight into the dynamic structure of phytoplankton communities in these states. Although our approach allowed us to study causal effects of non-linear hydrological patterns on phytoplankton communities that are relevant for understanding ecohydrological processes in wetlands [60,61], our approach limited an assessment of non-linear processes triggering phytoplankton changes between the wet and dry state. Notwithstanding, our approach shows how community dynamics can be inferred under contrasting ecological conditions and is therefore also suitable for assessing the relative resilience of alternative states in ecosystems and other complex systems.

 Given the strong impacts of hydrological disturbance on floodplain communities [62], it may not be surprising that phytoplankton dynamics differed between wet and dry states, thereby supporting also our hypothesis. We found that RDA models for the dry state did not select AEM variables indicating short-term fluctuations, suggesting that slower dynamics increase in relative importance in the dry relative to the wet state. Because the time series models were constructed from 24 time steps (months) for both states the differences observed are not confounded by unequal sampling frequencies and lengths of study periods. Jiang et al. [63] have shown that patterns of cyclic dynamics differ between alternative states, and that these dynamics emerge through biological variables (e.g., priority effects). Although our observational study precludes an assessment of biological interaction as the mechanism shaping the fluctuation patterns, abiotic factors cannot be ruled out. In the absence of flood pulsing, droughts have been considered as a disturbance that slowly increases in magnitude over time (i.e., a ramp disturbance) [24], influencing communities through decreased habitat availability, increasing heat stress, altered trophic conditions and resource competition [25]. Thus, the slowed dynamics during drought might arise from complex community responses to slow external factors (e.g. heat) rather than recurrent, seasonal flood disturbance. 

 Regarding resilience, model analysis has shown that slower dynamics can have a stabilizing effect of transitional dynamics and retard the return to stable alternative states following disturbance [64]. While this suggests that slow dynamics can increase resilience, we acknowledge that resilience is characterized by many attributes [41], and our study shows that several of these attributes of resilience should be assessed simultaneously to increase inference. Our time series modeling approach not only allowed assessing dominant fluctuation patterns of phytoplankton but also how patterns were compartmentalized by scale, a critical attribute of resilience [11]. In turn, this helped us evaluate the role of species richness mediating resilience.

 Increased resilience has been associated with a higher species richness and diversity in communities [19,20]. Species richness and diversity were higher during the dry compared to the wet state in this study [40]. However, our time series models suggest that resilience in the dry state may not have been necessarily higher. While the within-scale aspect of resilience did not show any clear differences between the wet and the dry state because the number of species at each scale was similar across the models, we found clear differences in the cross-scale structure. The number of dominant fluctuation patterns of phytoplankton or temporal scales was lower in the dry relative to the wet state. Theory and empirical studies have related resilience to the number of scales present in a system [12,65,66], assuming that resilience is increased with a higher cross-scale structure, thereby contributing to strengthen feedbacks through a stronger reinforcement of processes across scales. Because the number of temporal scales was reduced during the drought relative to the wet state, we can interpret this as a decreased cross-scale reinforcement of dynamics and thus lower resilience of phytoplankton communities in the dry state. Not only is this decreased resilience in agreement with the interpretation that drought comprises a perturbation for ecological communities [26,27], it also highlights a paradox: increased species richness may not necessarily increase resilience through a cross-scale reinforcement of patterns. This paradox can be further scrutinized with the number of species with stochastic dynamics identified by the time series models. 

 The number of species with apparently stochastic dynamics was on average higher in the dry compared to the wet state. Although inference is limited in our study because we could only compare a few sites, our results are consistent with other studies that have shown an increased component of stochastic community assembly during drought [26,27]. Also, the lower explanatory power of some statistical models of the dry state, highlighting increased stochasticity, is in agreement with this interpretation. If resilience is apparently reduced in the dry state, which role do species with stochastic patterns play in alternative states? 

 With the exception of a single study within an engineering resilience context [67], the role of stochastic processes in resilience research has not been explored. Stochastic patterns may be found in some rare taxa (with low abundances). Numerically rare species may have a significant role as they may increase in abundance following disturbance and thereby sustain important functions when dominant species are removed or novel conditions introduced [68]. However, the importance of rare species is easy to overlook, because the strength of ecological patterns is related to more dominant species. In time series models, the within and cross scale patterns identified are comprised of the temporal dynamics of these dominant species. However, when environmental conditions change, i.e. after regime shifts when ecosystems reorganize in alternative system states, rare species with apparently stochastic dynamics may become abundant and determine the within and cross scale structures in the new state. Thus, species with stochastic dynamics may not contribute to resilience *per se* in a specific state by means of a within and cross scale reinforcement of patterns but rather provide another critical component that influences the capacity of reorganization (i.e. adaptive capacity). Because our aims were to assess within and cross-scale structures mediating resilience a quantification of the adaptive capacity facet was beyond the scope of this study. However, our study makes clear how the role of species richness can be scrutinized if partitioned into patterns that reflect both the dynamic system structure at different scales and random noise. These patterns can be further explored for gaining a more process-based understanding of the role of species when ecosystems and communities re-organize in alternative states. 

 We conclude by highlighting the implications of our results for assessing resilience of ecological systems. The distribution and redundancies of functional attributes of species within and between scales or the capacity of organisms within functional groups to respond to disturbance (response diversity) critically mediate the overall resilience of ecological systems [11,12,69]. Assessing these functional distributions will require a sound estimate of the underlying scale-specific structure related to species distributions. The use of multivariate time series modelling is straightforward because it makes rates of environmental change at distinct temporal scales tractable, making possible inference regarding the relative resilience of ecological systems from a dynamic perspective. There is concern that current rates of anthropogenic impact will increase the incidence and frequency of regime shifts in ecological and combined social-ecological systems, with many of the new states providing fewer goods and services to humanity [19,37]. Using phytoplankton community dynamics in wet and dry states of a floodplain, our results show that the dynamic system structure necessary for understanding resilience can differ substantially between states. This highlights the usefulness of time series modeling to infer the relative resilience of alternative states across ecological systems and other complex systems with known histories of regime shifts when adequate time series data are available for analysis. 

## Supporting Information

Appendix S1 **Assessment of alternative wet-dry states in the wetland.**
(DOCX)

Appendix S2 **Flow chart outlining the steps involved in time series modeling.**
(DOCX)

Appendix S3 **Asymmetric Eigenvector Maps (AEM) variables selected by Redundancy Analysis.**
(DOCX)
