# Supplementary material for: Inferring the Relative Resilience of Alternative States
Source: PLoS One. 2013 Oct 11;8(10):e77338. doi: 10.1371/journal.pone.0077338 (PMC3795661; doi:10.1371/journal.pone.0077338)
Supplement: Appendix S1 — Assessment of alternative wet-dry states in the wetland. (DOCX) [file pone.0077338.s001.docx]

*Appendix S1*

Angeler et al.: Inferring the relative resilience of alternative states

Assessment of alternative wet-dry states in the wetland

We formally tested for thresholds in the time series to assure that phytoplankton community dynamics have been studied in contrasting (alternative) hydrological (wet-dry) states. The method used was based on the STARS algorithm [1,2]. The STARS software, which is freely available as an Excel macro, searches for shifts in the level of a univariate stationary time series by performing t-tests on individual observations, with the null hypothesis that the n^th^ observation is drawn from the same population as the preceding sequence of observations. If the null hypothesis of no shift is initially rejected, follow-up tests are performed on a specified number of subsequent observations. Models with different parameter settings showed congruent patterns of the regime shift; we therefore present models that have been obtained with default parameter settings [l (cutoff length) = 10, a (nominal size of test) = 0.05, h (Huber weight parameter) = 1].

We assessed changes in the wetlands hydrology using a single variable (flooded area), which is a conservative approach because multiple meteorological and hydrological variables interplay in the hydrological functioning of a wetland [3]. However, previous studies have shown that flooded area in the wetland is highly correlated with other hydrological variables (e.g., evapotranspiration [4], infiltration [5]), and that flooded area was adequate for studying plankton processes under different hydrological conditions in the wetland [6-9]. We therefore regard flooded area as an adequate proxy of the overall hydrological conditions in the wetland, and thus for assessing the temporal structure of phytoplankton communities in contrasting abiotic states.

1. Rodionov SN (2004) A sequential algorithm for testing climate regime shifts. Geophysical Reseaerch Letters 31: L09204.

2. Rodionov SN, Overland JE (2005) Application of a sequential regime shift detection method to the Bering Sea ecosystem. ICES Journal of Marine Sciences 62: 328–332.

3. Mitsch WJ, Gosselink, JG (2000) Wetlands, 3rd edn: Wiley & Sons.

4. Sánchez-Carrillo S, Angeler DG, Sánchez-Andrés R, Alvarez-Cobelas M, Garatuza J (2004) Evapotranspiration in semiarid wetlands: relationships between inundation and the macrophyte cover:open water ratio. Advances in Water Resources 27: 643-655.

5. Navarro V, García B, Asensio L (2012) Characterization of the infiltration rate in Las Tablas de Daimiel National Park, Central Spain. Hydrological Proccesses 26: 367-378.

6. Angeler DG, Alvarez-Cobelas M, Rojo C, Sánchez-Carrillo S (2000) The significance of water inputs to plankton biomass and trophic relationships in a semi-arid freshwater wetland (central Spain). Journal of Plankton Research 22: 2075-2093.

7. Angeler DG, Alvarez-Cobelas M, Rojo C, Sánchez-Carrillo S (2010) Phytoplankton community similarity in a semiarid floodplain under contrasting hydrological connectivity regimes. Ecologial Research 25: 513-520.

8. Rojo C, Ortega-Mayagoitia E, Rodrigo MA, Álvarez-Cobelas M (2000) Phytoplankton structure and dynamics in a semiarid wetland, the National Park “Las Tablas de Daimiel” (Spain). Archiv für Hydrobiologie 148: 397-419.

9. Rojo C, Álvarez-Cobelas M, Benavent-Corai J, Barón-Rodríguez MM, Rodrigo MA (2012) Trade-offs in plankton species richness arising from drought: a long-term study in a National Park wetland (Central Spain). Biodiversity and Conservation 21: 2453-2476.
