# Supplementary material for: Inferring the Relative Resilience of Alternative States
Source: PLoS One. 2013 Oct 11;8(10):e77338. doi: 10.1371/journal.pone.0077338 (PMC3795661; doi:10.1371/journal.pone.0077338)
Supplement: Appendix S3 — Asymmetric Eigenvector Maps (AEM) variables selected by Redundancy Analysis. (DOCX) [file pone.0077338.s003.docx]

*Appendix S3*

Angeler et al.: Inferring the relative resilience of alternative states

AEM variables selected by RDA

**
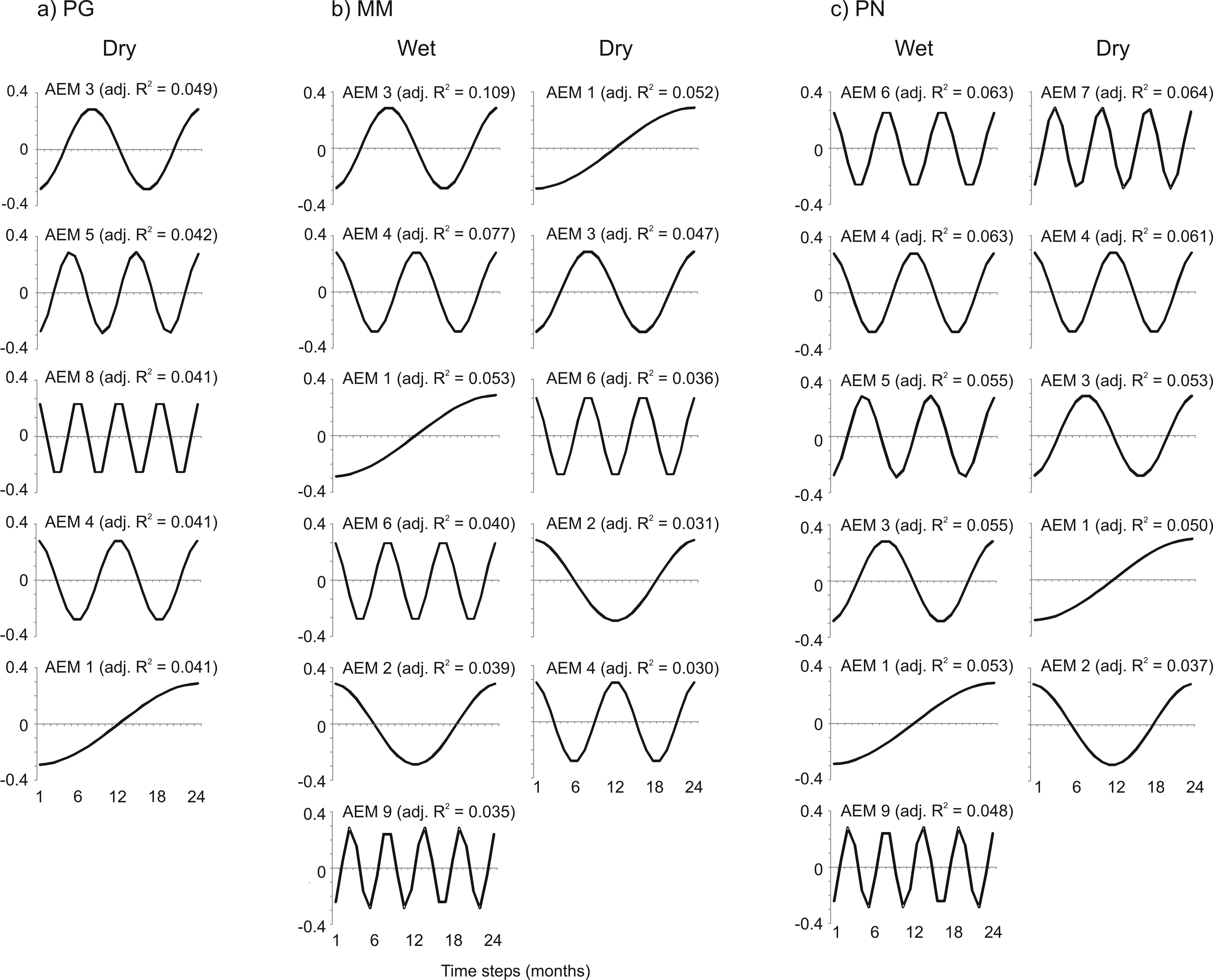
**

Significant AEM variables, reflecting different fluctuation frequencies, explaining the modeled temporal structure across study sites (a, PG; b, MM, c, PN) in the wet and dry states. The explanatory power of each axis (adjusted R^2^) that shows the relative importance of an individual frequency pattern for the temporal dynamics of phytoplankton is given in parentheses.
